# Sleep Apnea Pathophysiology in Patients with a History of COVID-19

**DOI:** 10.3390/jcm15020580

**Published:** 2026-01-11

**Authors:** Yeliz Celik, Scott A. Sands, Raichel Alex, Yüksel Peker, Susan Redline

**Affiliations:** 1 Koc University Research Center for Translational Medicine (KUTTAM), Koc University, Istanbul 34450, Turkey; yecelik@ku.edu.tr; 2 Division of Sleep and Circadian Disorders, Brigham and Women’s Hospital and Harvard Medical School, Boston, MA 02115, USA; sasands@bwh.harvard.edu (S.A.S.); ralex@bwh.harvard.edu (R.A.);; 3 Department of Pulmonary Medicine, School of Medicine, Koc University, Istanbul 34450, Turkey; 4 Department of Clinical Sciences, Respiratory Medicine and Allergology, Faculty of Medicine, Lund University, 22100 Lund, Sweden

**Keywords:** COVID-19, obstructive sleep apnea, physiological traits, arousal threshold, loop gain, upper airway collapsibility

## Abstract

**Background**: Emerging evidence suggests that COVID-19 may influence obstructive sleep apnea (OSA) pathophysiology by affecting upper airway collapsibility, ventilatory control, and arousal responses, raising the possibility of a bidirectional relationship. This study examined whether individuals with a history of COVID-19 show altered OSA-related physiological traits compared with those without prior infection. Methods: In a case–control study, 60 participants with a history of COVID-19 were compared to 60 matched controls who underwent overnight in-hospital polysomnography before the pandemic. The matching criteria included age (±5 years), gender, body mass index (BMI) (±5 kg/m^2^), and OSA presence. Key pathophysiological traits (collapsibility, loop gain, arousal threshold, muscle compensation) estimated from polysomnographic signals were compared, with adjustment for age, sex, BMI, and apnea–hypopnea index. **Results**: The participants (78% male, mean age 55 ± 12 years, BMI 29.4 ± 5.0 kg/m^2^) exhibited no meaningful differences in their average levels of collapsibility (Adj dif [95% CI]; *V_passive_*: −1 [−4, 2] %_eupnea_, *p* = 0.7), loop gain (*LG*_1_: 0.01 [−0.04, 0.06], *p* = 0.7), or arousal threshold levels (−1 [−7, 4] %_eupnea_) and showed similar levels of muscle compensation (*V_comp_*: 5 [−1, 11], *p* = 0.12). However, a greater ventilatory response to arousal (7 [1, 12] %_eupnea_) was associated with COVID-19 history. **Conclusions**: COVID-19 history is not associated with differences in key OSA pathophysiological traits, suggesting it is unlikely to explain observed differences in OSA presentation. The increased ventilatory response to arousal may have implications for treatment responses and outcomes.

## 1. Introduction

Beyond its acute effects, Coronavirus Disease 2019 (COVID-19) has been linked to a range of persistent symptoms, including fatigue, sleep disturbances, and respiratory dysfunction, that continue to affect a substantial number of individuals months after infection [1,2]. These overlapping features raise important questions about the relationship between COVID-19 and obstructive sleep apnea (OSA) [3,4], a common disorder characterized by upper airway collapse and disrupted breathing during sleep [5]. While OSA has been associated with worse outcomes during acute COVID-19 [6], there is growing interest in whether COVID-19 itself may influence the development or severity of OSA through lasting physiological changes [3]. Although a bidirectional relationship between COVID-19 and OSA has been proposed, the present study specifically focuses on whether a history of COVID-19 alters the physiological traits underlying OSA.

While OSA likely contributes to adverse outcomes of COVID-19, there are also mechanistic reasons why COVID-19 could influence OSA, as part of a bidirectional relationship. Pathophysiologically, COVID-19 may exacerbate upper airway collapsibility through nasal congestion-related pharyngeal collapse and snoring vibrations, which could feasibly persist beyond initial infection [7]. COVID-19 was also thought to cause a range of neurological effects, with reports of silent hypoxemia characterized by low oxygen saturation without dyspnea, with one study employing breath-hold testing, suggesting reduced ventilatory responsiveness in more severe cases [8]. Persistent viral brain-stem effects could theoretically damp multiple neurophysiological reflexes that may influence OSA pathophysiology, namely pharyngeal dilator muscle reflexes, the control of breathing (loop gain), or arousability (i.e., arousal threshold). On the other hand, residual lung damage post-COVID may exacerbate oxygen desaturation during apneas or hypopneas [9], potentialy augmenting chemoreflex responses (loop gain) through adaptative mechanisms. Despite these plausible mechanisms, data directly linking COVID-19 history to OSA physiological traits are lacking.

Accordingly, to assess whether post-COVID-19 patients exhibit distinct alterations in key OSA-related physiological traits, we examined OSA traits in participants who underwent in-hospital attended polysomnography (PSG) [10,11] and compared these with data from patients who underwent PSG testing before the COVID-19 pandemic. Specifically, we tested whether a history of COVID-19 was associated with differences in upper airway collapsibility, muscle compensation, ventilatory control (loop gain), and arousal threshold using an adjusted case–control analysis.

## 2. Materials and Methods

### 2.1. Study Population and Participants

**Figure 1:**
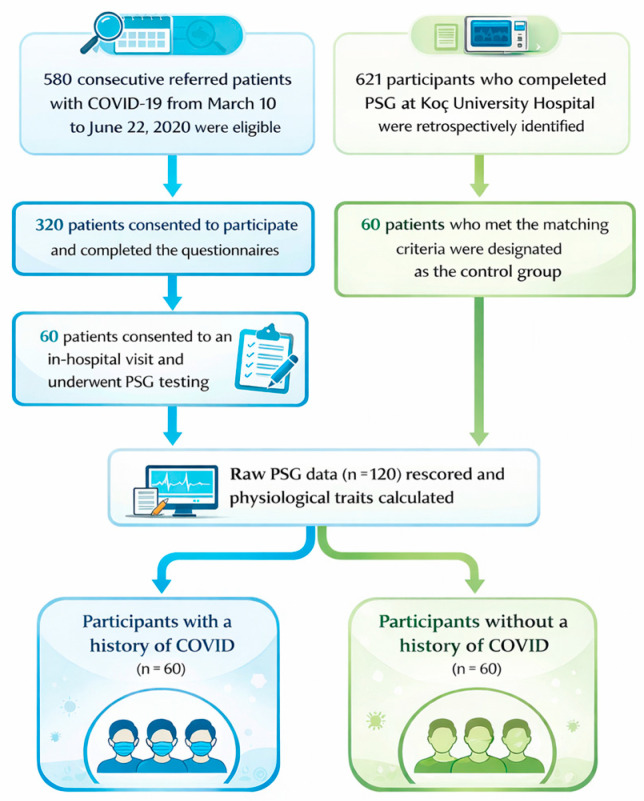
Overview of participant inclusion and final study groups.

As presented in Figure 1, this study included 60 participants from an original cohort of 320 individuals who were diagnosed with COVID-19 at Koç University Hospital and Koç Group American Hospital between 10 March and 22 June 2020 and who had participated in the initial study [11]. All 320 participants were subsequently recontacted and invited to participate in a follow-up study involving sleep-related questionnaires and overnight attended in-hospital PSG [10]. Of those invited, 60 individuals consented and successfully completed the full protocol; their data were used in the present analysis. The PSG assessments were conducted between 1 May and 31 October 2021. The eligibility criteria used in the initial study were as follows: (1) an age ≥ 18 years; (2) a confirmed diagnosis of COVID-19, defined by a positive polymerase chain reaction (PCR) test of nasopharyngeal specimens and/or clinical symptoms with radiologic findings consistent with COVID-19 pneumonia, such as ground-glass opacities with a peripheral and subpleural distribution or focal consolidation; (3) the ability to read and speak; and (4) the provision of written informed consent.

For the control group, 60 individuals were retrospectively identified from the Koç University Hospital Sleep Laboratory database. AHI was not included in the matching criteria, as it was a primary outcome of interest. Including AHI as a matching variable could have led to overmatching, potentially obscuring differences in sleep traits that may have emerged following COVID-19 infection and hospitalization-related hypoxic exposure, and thereby limiting our ability to detect meaningful differences in OSA severity between the groups. These participants had undergone PSG prior to the COVID-19 pandemic. Selection criteria for the control group included matching age (±5 years), body mass index (BMI) (±5 kg/m^2^), and gender in a 1:1 ratio [12]. This design allowed us to directly assess whether prior COVID-19 was associated with increased severity of obstructive sleep apnea. All of the ethical approvals for this study were obtained from the Koç University Institutional Review Board (IRB) and written informed consent was obtained from all participants. The inclusion of patients with a documented history of COVID-19 was approved under IRB protocol 2021.231.IRB2.049. The access to and use of clinical records for the matched non-COVID control cohort were approved based on the institutional authorization for the TURKAPNE study, formally granted by Koç University Hospital on 25 September 2019. The overall study design, data integration procedures, and final analytic plan for the present manuscript were reviewed and approved under IRB protocol 2024.048.IRB2.022. All procedures complied with the Declaration of Helsinki and applicable national regulations.

### 2.2. Sleep Measurements

In this study, full-night PSG was conducted at the Koç University Hospital Sleep Laboratory using the NOX-A1 system (Nox Medical Inc., Reykjavik, Iceland). The attended PSG included recordings of electroencephalography (EEG), electrooculography (EOG), chin and leg electromyography (EMG), snoring intensity, nasal airflow, thoraco-abdominal and leg movements, body position, heart rate, oxyhemoglobin saturation (SpO_2_), and video monitoring of participants.

To minimize potential temporal or scoring-related differences, all of the polysomnography recordings from both groups were reanalyzed using the same standardized MATLAB (version R2023a, The MathWorks, Inc., Natick, MA, USA) -based algorithms to derive physiological trait estimates. Sleep stages and arousals were scored in 30 s epochs according to criteria established by the American Academy of Sleep Medicine (AASM) [13]. Apnea was defined as a nearly complete (≥90%) cessation of airflow, while hypopnea was identified as a ≥30% reduction in nasal pressure amplitude and/or thoraco-abdominal movement lasting at least 10 s, accompanied by either a significant oxyhemoglobin desaturation (≥3% reduction from baseline) or an arousal [13].

In addition to the total number of significant desaturations, the oxygen desaturation index (ODI) was calculated as the number of desaturation events (≥3%) per hour of total sleep time. The time spent with SpO_2_ below 90% (TS90%) and the minimum SpO_2_ values were also recorded.

### 2.3. Physiological Trait Calculations

Four physiological traits were extracted from the PSG data using the methodology described by Sands and colleagues [14,15,16,17]. To quantify these traits, the PSG data were divided into 7 min windows. Ventilation was estimated from nasal pressure signals, where 0% indicated complete apnea and 100% corresponded to eupneic breathing. The nasal pressure signal was used as a surrogate for ventilatory flow and was integrated to produce a breath-by-breath ventilation signal, calculated as the product of uncalibrated tidal volume and respiratory rate. Within each 7 min segment, ventilatory drive, which reflects the intended ventilation that would occur in the absence of airway obstruction, was estimated using a chemoreflex feedback control model. Ventilatory drive was calculated by applying the ventilation signal to a simplified chemoreflex feedback control model that included unknown parameters such as loop gain, response time, and delay. The model output represented the ventilatory (chemoreflex) drive and the parameters were optimized through a least-squares approach to best fit the estimated ventilatory drive signal to the actual ventilation signal during obstructive events. This modeling process was conducted separately for each 7 min window containing sleep.

Loop gain was derived from the model for each 7 min window and evaluated using two parameters. The LG_1_ (primary measure) represented the sensitivity of the ventilatory feedback loop, describing the magnitude of the ventilatory drive response to a 1 cycle/min disturbance. The LGn measured the overall ventilatory instability by incorporating the effects of chemoreflex sensitivity and circulatory delay. A value of LGn greater than 1 indicated cyclic central sleep apnea.

For each 7 min window, the ventilatory drive immediately preceding a scored EEG arousal was identified, and the arousal threshold was calculated as the average ventilatory drive at these time points. A single median value across all windows was obtained for each subject. Lower arousal threshold values indicated greater arousability from sleep, while higher values reflected reduced responsiveness to respiratory stimuli. Additionally, the ventilatory response to arousal (VRA), which quantifies the increase in ventilatory drive triggered by arousal and is independent of chemical drive, was also determined.

Pharyngeal collapsibility was characterized using two parameters: passive collapsibility (Vpassive) and active collapsibility (Vactive). Vpassive (primary measure) was defined as the ventilation at normal (eupneic) ventilatory drive, with lower values indicating a more collapsible airway. Vactive, which represented ventilation at the arousal threshold, reflected the maximum activation of the upper airway. Pharyngeal muscle compensation was calculated as the difference between Vactive and Vpassive, where higher values indicated stronger compensatory muscle activity.

The entire analysis was automated using in-house software (Phenotyping Using Polysomnography) developed in MATLAB (MathWorks). All of the analytical steps were visually inspected by YC.

### 2.4. Statistical Analysis

The descriptive statistics for the sleep parameters and physiological traits were reported as a median with an interquartile range (25–75%) due to the data distribution. Multiple linear regression models estimated the group differences in physiological traits adjusting for age, gender, BMI, and AHI. The primary measures were the passive collapsibility (V_passive_), muscle compensation, loop gain (LG_1_), and arousal threshold. The adjusted group differences in additional traits that met the *p* < 0.05 criteria were reported but considered exploratory. To evaluate the robustness of our findings, a sensitivity analysis was also conducted in a subgroup of participants matched on AHI (±5 events/h), in addition to age, gender, and BMI, using the same regression approach. The data processing and statistical analyses were performed using IBM SPSS 28.0 for Windows (IBM Corp., Chicago, IL, USA).

## 3. Results

### 3.1. Baseline Sleep Characteristics of the Study Groups

In the overall study population, 78.3% of participants were male, with a mean age of 55 ± 11.8 years. The mean BMI was 29.4 ± 5.0 kg/m^2^ and 43% of the participants were classified as obese. The distribution of AHI across the study sample (N= 120) was right-skewed, with a mean (SD) of 14.6 (18.5) events/h and a median of 7.9 events/h. AHI values ranged from 0.0 to 88.4 events/h. In an unadjusted analysis (Table 1), individuals with a history of COVID-19 infection exhibited less severe OSA, as indicated by a lower total AHI. We also noted a lower arousal index, slightly lower Stage 1 sleep duration, and higher REM sleep percentages, consistent with their lower OSA severity.

### 3.2. Comparison of Physiological Trait Measurements Across Study Groups

The distribution of physiological traits between participants with and without a history of COVID-19 infection is presented in Table 2. Crude inspection of the group distributions suggested less collapsible upper airways and greater pharyngeal muscle compensation compared to those without infection, as well as a lower arousal threshold, in unadjusted comparisons.

In the primary adjusted analysis, however, we found no meaningful differences in upper airway collapsibility (difference in *V_passive_*: −1 [−4, 2] %_eupnea_, *p* = 0.7), loop gain (difference in *LG*_1_: 0.01 [−0.04, 0.06], *p* = 0.7), and arousal threshold levels (difference: −1 [−7, 4] %_eupnea_). Pharyngeal muscle compensation also showed no significant group differences (Table 2), despite a small increase in compensation (difference: 5 [−1, 11]) towards less deleterious values in those with a history of COVID-19.

The only sign of a deleterious trait was the observation of a higher ventilatory response to arousal (7 [1, 12] %_eupnea_) observed in association withCOVID-19 history in adjusted analyses (with similar levels prior to adjustment).

Overall, after adjustment, most physiological traits remained stable between groups, with ventilatory response to arousal representing the only trait that differed significantly between participants with and without a history of COVID-19.

### 3.3. Comparison of Physiological Traits in OSA Patients

In an exploratory analysis restricted to patients with OSA (AHI ≥ 5 events/h), findings were largely consistent (Table 3): The 6% increase in the ventilatory response to arousal in the COVID-19 group remained persistent (albeit with reduced significance). A physiologically meaningful increase in muscle compensation in the COVID-19 group of +8 [−2, 17] %_eupnea_ became somewhat clearer, but was not significant.

### 3.4. Sensitivity Analysis in AHI-Matched Subgroup

To address reviewer concerns regarding matching strategy and to determine whether differences in OSA severity influenced our primary findings, we performed a sensitivity analysis in a subgroup of participants with a history of COVID-19 (n = 40) and matched controls (n = 40) whose AHI values fell within ±5 events/h. As expected, the AHI did not differ between groups (*p* = 0.922), confirming successful severity matching. In this AHI-matched subset, most physiological traits, including passive and active collapsibility, loop gain, arousal threshold, ventilatory instability, delay, and VRA, did not differ significantly between groups (Supplementary Table S1). The only significant difference observed was greater muscle compensation in individuals with prior COVID-19 (adjusted difference: 5% [95% CI: 1.96–9.09], *p* = 0.01), indicating that COVID-19 patients exhibited better neuromuscular compensation of the upper airway.

## 4. Discussion

The current study investigated whether key pathophysiological traits contributing to obstructive sleep apnea differ between individuals with a history of COVID-19 infection and matched controls studied before the pandemic. We did not observe differences in the four primary physiological traits underlying OSA, namely pharyngeal collapsibility, loop gain, arousal threshold, and muscle compensation, indicating that endotypic mechanisms are unlikely to differ according to COVID-19 history. However, an increased ventilatory response to arousal was observed in the adjusted analyses, which may modestly influence ventilatory instability and arousal responses during sleep.

To address concerns regarding the matching strategy, a sensitivity analysis that was also matched for AHI was performed and confirmed there were no meaningful differences in physiological traits between groups. These findings support the decision not to include AHI in the primary matching procedure, as matching on AHI could have resulted in overmatching and obscured potential trait-level differences associated with COVID-19 history.

Observational studies have shown a strong association between pharyngeal collapsibility and clinical manifestations of OSA, with individuals diagnosed with OSA exhibiting greater airway collapsibility than age-, sex-, and BMI-matched controls, both under general anesthesia and during sleep [18,19]. In the current study, we considered the possibility that upper airway collapsibility might be milder in patients with a history of COVID-19, particularly in cases where elevated loop gain played a more dominant role in their OSA pathophysiology or when such patients experienced greater oxygen desaturation for the same level of airway compromise due to residual gas exchange abnormalities. However, our findings indicate that upper airway collapsibility was virtually identical in the adjusted analysis to values seen in patients without a history of COVID-19.

The activation of pharyngeal dilator muscles is essential for maintaining pharyngeal patency and ventilation during sleep, yet its responsiveness varies significantly among individuals with OSA [20]. Moreover, the extent to which increased dilator muscle activation resolves airway compromise is highly heterogeneous [21]. Given possible adverse long term neurological effects of COVID-19 infection, we expected to find reduced dilator muscle activation in individuals with a history of COVID-19. However, in the present study, we observed no difference between groups; if anything, there was a tendency towards greater muscle compensation in individuals with a history of COVID-19. Accordingly, our findings imply that it is highly unlikely that reduced muscle compensation is a mechanism by which a history of COVID-19 might promote OSA.

Loop gain, a measure of ventilatory control stability, is involved in the pathophysiology of OSA [22,23] and we considered that it could be altered in patients with a history of COVID-19 due to hypoxic exposure or potential neural effects on respiratory chemoreflex control [24]. However, in our adjusted analysis, loop gain values were similar between the COVID and non-COVID groups, indicating that COVID-19 history is not a likely explanation for any observed increases in ventilatory instability in clinical practice.

We considered that prior exposure to hypoxia during acute COVID-19 may have led to a higher arousal threshold, potentially prolonging airway obstruction by delaying respiratory event termination [25]. We found no differences in arousal threshold between individuals with and without a history of COVID-19, suggesting that clinically observed increases in arousability after COVID-19 are unlikely to be a direct consequence of prior infection. This null finding carries important implications: Specifically, given the associations between low arousal threshold and low CPAP adherence [26], a history of COVID-19 is unlikely to affect CPAP adherence via its impact on arousal threshold. Likewise, the efficacy of hypoglossal nerve stimulation therapy, found to be less effective in patients with a low arousal threshold [27], is unlikely to be influenced by a prior history of COVID-19. For patients with high arousability, our data suggests that alternative explanations, such as post-viral inflammation, autonomic dysregulation, or psychological factors including PTSD, should be considered to explain increased arousability in OSA patients with a history of COVID-19.

Beyond the four key OSA pathophysiological traits, the current study found an increase in the ventilatory response to arousal in association with COVID-19 history in an exploratory analysis. An augmented ventilatory response to arousal is considered destabilizing from the perspective of ventilatory control [28], raising the likelihood of a future respiratory event by lowering ventilatory drive stimuli that are necessary for maintaining airway patency in patients with a vulnerable airway. Indeed, a higher ventilatory response to arousal may be a predictor of the reduced treatment efficacy of oral appliances [29]. A greater ventilatory arousal response may also indirectly promote increased heart rate and arousal [30] effects, which may have implications for adverse cardiovascular or neurocognitive consequences of OSA [31]. Further research is needed to replicate this association and better elucidate its clinical implications.

An increased ventilatory response to arousal may also have important clinical implications in patients with a history of COVID-19. Heightened arousal-related ventilation could contribute to greater ventilatory instability and sleep fragmentation, potentially influencing OSA phenotypes characterized by increased arousability or symptom burden. From a therapeutic perspective, such a response may affect treatment responsiveness, as elevated ventilatory overshoot has been associated with reduced efficacy of oral appliance therapy and may also modify responses to CPAP, supplemental oxygen, or pharmacological approaches targeting ventilatory control. Mechanistically, this finding may reflect persistent alterations in chemoreflex sensitivity or autonomic regulation following COVID-19, although given the exploratory nature of these analyses, further studies are required to confirm this association and clarify its clinical relevance.

Limitations: First, the control group was drawn from a sleep clinic cohort and had more severe OSA than participants with a history of COVID-19, which may have introduced selection bias despite matching for age, sex, and BMI. Participants were not matched by AHI because AHI was a primary outcome of interest and matching on AHI could have resulted in overmatching and masked meaningful differences in OSA-related physiological traits, although future studies may consider AHI matching to further isolate COVID-19 effects. In addition, pre-infection sleep studies were unavailable for the COVID-19 group, limiting our ability to determine whether OSA preceded or followed infection. Second, PSG assessments in the COVID-19 group were conducted approximately one year after infection and our findings may therefore reflect partial recovery or stabilization of OSA severity over time, as airway obstruction symptoms have been reported to be more prominent during the acute phase of upper airway infection [10]. Third, OSA pathophysiology was assessed using estimated physiological traits rather than gold-standard invasive measures, which may warrant further validation, although invasive approaches may be less tolerable and introduce bias in patients with increased arousability. Fourth, the study population was predominantly male (78%), which may limit the generalizability of the findings, particularly in light of known sex-related differences in obstructive sleep apnea pathophysiology.

## 5. Conclusions

The present study addresses the novel question of whether the physiological traits of OSA differ in individuals with a history of COVID-19 infection compared to controls. We found no associations between COVID-19 history and any of the four key traits when adjusting for age, sex, BMI, and AHI. Given the overall similarity of physiological traits, our findings do not suggest the need to modify the current treatment approaches for OSA in patients with prior COVID-19 infection.

## Figures and Tables

**Table 1:** Participants’ polysomnographic characteristics.

	History of COVID-19 n = 60	Controls n = 60	*p*
TST, min	380.2 (349.1–406.8)	315.7 (272.7–365.8)	<0.001
WASO, min	46.0 (25.5–82.5)	49.3 (27.6–84.5)	0.57
Stage 1, (%)	3.5 (2.2–5.3)	5.3 (3.6–8.2)	0.001
Stage 2, (%)	55.9 (49.1–61.8)	56.0 (48.8–63.9)	0.89
Stage 3, (%)	22.4 (17.2–26.7)	22.7 (15.9–28.6)	0.98
REM, (%)	17.5 (12.5–20.3)	13.1 (10.1–0.18.0)	0.014
Arousal Index	39.11 (28.82–52.30)	47.49 (38.86–50.12)	0.003
AHI Events/h	13.5 (8.0–23.6)	19.3 (10.8–37.7)	0.004
AHI Non-REM	11.1 (6.3–22.7)	19.6 (10.9–39.1)	0.002
AHI REM	20.1 (8.2–33.0)	21.7 (8.2–33.0)	0.38
ODI	6.1 (2.8–13.5)	10.0 (3.1–24.8)	0.09
Average Desaturation	2.7 (2.1–3.8)	3.2 (2.0–4.5)	0.23
Average SpO2	93.4 (92.2–94.5)	93.3 (92.1–94.6)	0.72
Minimum SpO2	84.0 (80.3–87.0)	84.5 (78.3–88.0)	0.90
SpO2 Duration < 90 min	1.0 (0.2–6.7)	2.1 (0.1–9.0)	0.52

Continuous data are presented as medians with 25–75% quartiles. Categorical data are presented as counts with percentages. Abbreviations: AHI, apnea hypopnea index; ODI, oxygen desaturation index; REM, repeat eye movements; WASO, wake after sleep onset.

**Table 2:** Pathophysiological trait comparisons between individuals with and without a history of COVID infection.

	History of COVID-19 n = 60	Controls n = 60	Adjusted Difference: [95% CI]	*p*
Primary Traits				
Passive Collapsibility (Vpassive), %_eupnea_	97 (95–98)	95 (90–98)	−1 [−4, 2]	0.6
Muscle Compensation, %_eupnea_	4 (3–6)	4 (−4–5)	5 [−1, 11]	0.12
Loop Gain (LG_1_)	0.45 (0.39–0.57)	0.50 (0.39–0.61)	0.01 [−0.04, 0.06]	0.7
Arousal Threshold, %_eupnea_	103 (101–108)	106 (102–123)	−1 [−7, 4]	0.7
Additional Traits				
Active Collapsibility (Vactive), %_eupnea_	102 (100–104)	99 (86–103)	4.1 [−2.9, 10.9]	0.25
Ventilatory Instability (LGn)	0.36 (0.31–0.44)	0.40 (0.33–0.44)	0.01 [−0.03, 0.04]	0.6
Delay, s	12.5 (10.1–14.1)	11.6 (10.3–13.8)	0.2 [−0.8, 1.3]	0.7
VRA, %_eupnea_	22.4 (11.3–34.4)	23.0 (11.7–35.7)	6.6 [1.0, 12.3]	0.022

Continuous group data are presented as medians (25–75% quartiles). Primary traits: *Vpassive*: collapsibility under passive conditions, lower values reflect a more collapsible airway. Muscle compensation: Vactive minus Vpassive (reflects an increase in airflow due to the activation of pharyngeal dilator muscles). *Loop gain* (LG_1_): ventilatory control sensitivity, without chemoreflex delay effects included. Arousal threshold: level of estimated drive prior to arousal, lower values reflect greater arousability. Additional traits: *Vactive:* collapsibility under active conditions, lower values reflect a more collapsible airway. *Ventilatory instability (LGn)*: measure of loop gain that reflects overall ventilatory control stability (includes the effect of circulatory delay) and represents predisposition to central sleep apnea. *Delay*: chemoreflex latency, time between a reduction in ventilation and the onset of an opposing increase in ventilatory drive. *VRA*: ventilatory response to arousal, the additional (non-chemical) increase in ventilation that is attributable to the presence of arousal, rather than the prior drop in ventilation. The adjusted differences and accompanying *p* values represented were estimated using linear regression, adjusting for age, gender, BMI, and AHI.

**Table 3:** Pathophysiological trait comparisons restricted to an AHI ≥ 5 events/h.

	History of COVID-19 n = 36	Controls n = 41	Adjusted Difference: [95% CI]	*p*
Primary Traits				
Passive Collapsibility (Vpassive), %_eupnea_	95 (90–98)	93 (88.00–96.84)	1 [−5–4]	0.8
Muscle Compensation, %_eupnea_	4 (3–7)	1 (−13–5)	7.56 [−1.80–16.91]	0.11
Loop Gain (LG_1_)	0.46 (0.37–0.60)	0.53 (0.43–0.70)	−0.03 [−0.09–0.04]	0.5
Arousal Threshold, %_eupnea_	106 (102–117)	110 (103–129)	−2 [−10–5]	0.5
Additional Traits				
Active Collapsibility (Vactive), %_eupnea_	102 (95–104)	94 (66–101)	6.9 [−3.8–17.6]	0.20
Ventilatory Instability (LGn)	0.39 (0.33–0.44)	0.42 (0.35–0.47)	−0.004 [−0.05–0.04]	0.9
Delay, s	13.1 (10.4–14.2)	11.1 (9.9–13.2)	0.6 [−0.6–1.8]	0.33
VRA, %_eupnea_	26.8 (19.1–40.6)	27.4 (18.6–39.9)	6.2 [−1.6–14.1]	0.12

Continuous group data are presented as medians (25–75% quartiles). Primary traits: Vpassive: collapsibility under passive conditions, lower values reflect a more collapsible airway. Muscle compensation: Vactive minus Vpassive (reflects an increase in airflow due to activation of pharyngeal dilator muscles). Loop gain (LG_1_): ventilatory control sensitivity, without chemoreflex delay effects included. Arousal threshold: level of estimated drive prior to arousal, lower values reflect greater arousability. Additional traits: Vactive: collapsibility under active conditions, lower values reflect a more collapsible airway. Ventilatory instability (LGn): measure of loop gain that reflects overall ventilatory control stability (includes the effect of circulatory delay) and represents predisposition to central sleep apnea. Delay: chemoreflex latency, time between a reduction in ventilation and the onset of an opposing increase in ventilatory drive. VRA: ventilatory response to arousal, the additional (non-chemical) increase in ventilation that is attributable to the presence of arousal, rather than the prior drop in ventilation. The adjusted differences and accompanying *p* values represented were estimated using linear regression, adjusting for age, gender, BMI, and AHI.

## Data Availability

The data that support the findings of this study are available upon reasonable request. Interested researchers may obtain access by sending an email to the corresponding author at yecelik@ku.edu.tr. Requests will be reviewed and shared when appropriate.

## Supplementary Materials

The following supporting information can be downloaded at: https://www.mdpi.com/article/10.3390/jcm15020580/s1, Table S1: Comparison of physiological traits in a subgroup of participants matched on AHI (±5 events/h), age, BMI, and gender.
