# Supplementary material for: Sleep Apnea Pathophysiology in Patients with a History of COVID-19
Source: J Clin Med. 2026 Jan 11;15(2):580. doi: 10.3390/jcm15020580 (PMC12842427; doi:10.3390/jcm15020580)
Supplement: Supplementary file 1 [file jcm-15-00580-s001.zip › jcm-4032546-supplementary.pdf]

**Supplementary Table S1.** Comparison of physiological traits in a subgroup of participants matched on AHI (±5 events/hour), age, BMI, and gender.

| | History of COVID-19 n = 40 | Controls n = 40 | Adjusted Difference: [95% CI] | *p* |
|---|---|---|---|---|
| **Primary Traits** | | | | |
| Passive Collapsibility (Vpassive), $\%_{eupnea}$ | 97.4 (95–98.6) | 96.6 (93.2–98.2) | 0.7 [−1.3–2.7] | 0.50 |
| Muscle Compensation, $\%_{eupnea}$ | 4 (3–7) | 4 (1.8–5.1) | 5 [1.96–9.09] | 0.01 |
| Loop Gain (LG1) | 0.46 (0.37–0.60) | 0.4 (0.3–0.55) | 0.03 [−0.02–0.09] | 0.19 |
| Arousal Threshold, $\%_{eupnea}$ | 103 (100–107) | 102 (101–108) | −2.3 [−7–2.3] | 0.32 |
| **Additional Traits** | | | | |
| Active Collapsibility (Vactive), $\%_{eupnea}$ | 102 (99–103) | 100 (93–104) | 6.8 [−0.6–14.3] | 0.15 |
| Ventilatory Instability (LGn) | 0.39 (0.32–0.44) | 0.36 (0.30–0.43) | −0.034 [−0.01–0.08] | 0.104 |
| Delay, s | 12.7 (10.1–13.6) | 12.2 (19.9–15.1) | 0.16 [−1.3–1.5] | 0.80 |
| VRA, $\%_{eupnea}$ | 20.5 (11.1–30.8) | 20.1 (0.7–27.3.9) | 4.4 [−0.9–9.8] | 0.104 |

Continuous group data are presented as medians (25–75% quartiles). Primary traits: Vpassive: collapsibility under passive conditions, lower values reflect a more collapsible airway. Muscle compensation: Vactive minus Vpassive (reflects increase in airflow due to activation of pharyngeal dilator muscles). Loop gain (LG1): ventilatory control sensitivity, without chemoreflex delay effects included. Arousal threshold: level of estimated drive prior to arousal, lower values reflect greater arousability. Additional traits: Vactive: collapsibility under active conditions, lower values reflect a more collapsible airway. Ventilatory instability (LGn): measure of loop gain that reflects overall ventilatory control stability (includes the effect of circulatory delay) and represents predisposition to central sleep apnea. Delay: chemoreflex latency, time between a reduction in ventilation and the onset of an opposing increase in ventilatory drive. VRA: ventilatory response to arousal, the additional (non-chemical) increase in ventilation that is attributable to the presence of arousal, rather than the prior drop in ventilation. The adjusted differences and accompanying *p* values represented were estimated using linear regression, adjusting for age, gender, BMI, and AHI.
